# NK cell-based immunotherapy strategies for myeloid leukemia

**DOI:** 10.3389/fimmu.2025.1621885

**Published:** 2025-07-14

**Authors:** Lin Zhang, Yibo Zhao, Yan Dong, Xiuxing Jiang

**Affiliations:** ^1^ Department of Pharmacy, Daping Hospital, Army Medical University, Chongqing, China; ^2^ Department of Oncology, Southwest Hospital, Army Medical University, Chongqing, China; ^3^ Frontier Medical Training Brigade, Army Medical University, Xinjiang, China

**Keywords:** myeloid leukemia, NK cell, exhaustion, therapeutic potential, immunobiology

## Abstract

Myeloid leukemia (ML) is a clonal malignant disease with abnormal hematopoietic stem cells. With the emergence of novel immunotherapies, such as CAR-T, therapeutic outcomes in ML patients have improved, while significant challenges persist, including severe adverse events and disease recurrence. Natural killer cells (NK cells) are “natural killers” of the immune system that do not require antigen presentation and responsible for recognizing and destroying tumor cells. Some NK cells-based clinical experiments have been carried out and achieved remarkable results with lower side effects in ML. Crucially, within the ML microenvironment, NK cells frequently exhibit more severe functional exhaustion compared with T cells, characterized by impaired cytotoxicity, cytokine production, and proliferative capacity which limits anti-ML efficacy of NK cells. However, clinical studies utilizing NK cell-based therapies (e.g., adoptive transfer, CAR-NK cells) have demonstrated promising results with favorable safety profiles, underscoring their therapeutic potential. Therefore, developing more strategies based on NK cell is of great clinical significance for the treatment of ML. In this review, we systematically analysed the relationship between ML and NK cells, aiming to propose more novel protocols for NK cell expansion and persistence enhancement, establish evidence-based guidelines for next-generation NK cell-based immunotherapies in ML treatment.

## 1 Introduction

Hematological malignancies (HMs), including four subtypes (non-Hodgkin lymphoma (NHL), leukemia (with several subtypes), multiple myeloma (MM), and Hodgkin lymphoma (HL)), are the fourth leading cause of cancer-related deaths, which contribute approximately 7% of global cancer incidence with an upward trend in prevalence (1–3). Among HMs, leukemia is a hematologic neoplasm with the characteristic of the excessive production of immature or mature blood cells. Despite advances in understanding disease pathogenesis and therapeutic interventions, leukemia remains a significant global health burden, ranking as the 13th most common malignancy (2.4% of all cancer cases) and the 10th leading cause of cancer-related mortality (3.1%) in 2022 (1–3).

Haematopoiesis is governed by haematopoietic stem cells (HSCs) that produce all lineages of blood and immune cells (4). These HSCs maintain blood homeostasis through dynamic stress-response mechanisms, whose dysregulation can trigger leukaemia (4). Leukaemogenesis involves chromosomal abnormalities (5, 6), gene mutation (7), and immune system disorders in leukemia stem cells (LSCs) (8), leading to abnormal proliferation and differentiation of LSCs, principally in the BM, and disrupting normal hematopoietic genesis (9, 10). Leukemia is classified into myeloid leukemia (ML) and lymphocytic leukemia (LL) based on lineage commitment and cellular maturation (11). ML results from acquired driver and cooperating mutations within HSCs or myeloid progenitors (12). Acute myeloid leukemia (AML) is characterized by accumulation of 20% or more abnormal leukemic blast cells, principally in the bone marrow (BM), and impaired normal blood cell production, leading to anemia and thrombocytopenia (12). In contrast, chronic myeloid leukemia (CML) exhibits accumulation of abnormally mature leukocyte, with clinical feature such as severe blood granulocytosis, granulocytic immaturity, basophilia, frequent thrombocytosis, anemia, and splenomegaly (12). LL can originate from cells across a wide spectrum of stages of T-, NK-, or B-lymphocyte differentiation (13). While acute lymphocytic leukemia (ALL) arises from early lymphoid progenitors expressing pre-B or pre-T cell phenotypes (14), chronic lymphocytic leukemia (CLL) derives from a more mature B-lymphocyte progenitor, characterized by accumulation of apoptosis-resistant B-cells (15).

Mounting evidence demonstrates that the tumor microenvironment (TME), the complex cellular ecosystem in which malignant cells emerge, plays a pivotal role in cancer pathogenesis (16). Within secondary lymphoid organs (SLOs) and BM, the TME comprises a heterogeneous population of stromal cells, including fibroblasts, cells of the innate and adaptive immune response, and vascular endothelial cells (ECs) (16). NK cells, as potent cytotoxic effectors of innate immunity, serve as a primary surveillance system against leukemia transformation (17, 18).

Unlike T cells and B cells, NK cells are the “rapid reaction forces” in the immune system (19), exhibiting pronounced exhaustion phenotype during early-stage leukemic progression (20–22). CAR-NK cells have shown a relatively low incidence of off-target effect and CRS, almost no ICANS in clinical applications, significantly reducing the risk of GvHD (23–25). Emerging clinical evidence validates the therapeutic efficacy of CAR-NK cells in CD19^+^ B-cell leukemia (26), non-Hodgkin’s lymphoma or CLL (27), and MM (28). Therefore, NK cells may play a more direct and important role in tumorigenesis, ML treatment strategies based on NK cells would be an effective immunotherapy strategy. In this review, we will summarize NK cell immunobiology and potential targets or strategies for ML treatment systematically in the aspect of NK cell-based immunotherapy.

## 2 NK cell immunobiology in cancer

Both human and murine NK cells differentiate from HSCs in BM before migrating to peripheral tissues (29, 30). For murine NK cells, HSCs are lineage negative (Lin^-^) stem cells (31), which can differentiate into NK progenitor cells (NKPs) (32). Acquisition of CD122 is a crucial step in NK cell specification (33). After specification, NK cells sequentially acquire the expression of cytokine receptors (e.g., CD27, CD122, CD127, CD244) (34, 35), activation and inhibitory receptor, adhesion molecules (e.g., integrin) (36), and chemotactic receptors (37). The activation receptor, NKG2D,which is widely expressed on NK cells, serves as a critical determinant in the initial transition of NKPs into immature NK cells (iNKs) (38), while the characteristic of NK cells mature to the DX5^+^ stage is the acquisition of the Ly49 family receptors (39). The expression of CD43 or CD11b determines the ultimate maturation of NK cells via PYK-2 signaling or Src/β-catenin pathway (40, 41). Mature NK cells (mNKs) can acquire inhibitory Ly49 receptors, including Ly49A/C/I/G and NKG2A, which could attenuate NK cell responses to normal cells expressing MHC-I molecules, thereby enabling more robust responses to infected or cancerous cells lacking MHC-I on the cell surface (42–44).

Human NK cells develop along a continuum of progressively down-regulated CD34 and up-regulated CD56 in common lymphoid progenitor cells (CLPs) (45). HSCs differentiate into multipotent progenitor cells and then transform into CLPs (46). CLPs differentiate into NKPs through transcriptional regulation mediated by key factors including GATA2 and E4bp4 (47). And then, NKPs differentiate into iNKs (48), characterized by high expression of IL-1R1 (49) and the appearance of NKG2D (38), NKp30/46 (50, 51) and CD161 (52). The next stage is the emergence of CD56^bright^ NK cells exhibiting potent cytokine production (53, 54). Finally, CD56^bright^ NK cells transform into CD56^dim^ NK cells (55, 56), which have higher CD16 expression and cytotoxicity (57). NK cell subsets could be used to stratify patients for NK-based therapies. For example, for NK cells exhibiting CD16^+^ expression or elevated NKG2A^+^ NK cell infiltration in the TME, personalized therapeutic approaches may include: combination with monoclonal antibodies (e.g., anti-CD20) (58) or co-administration with NKG2A inhibitors (e.g., monalizumab) (59).

**Figure 1:**
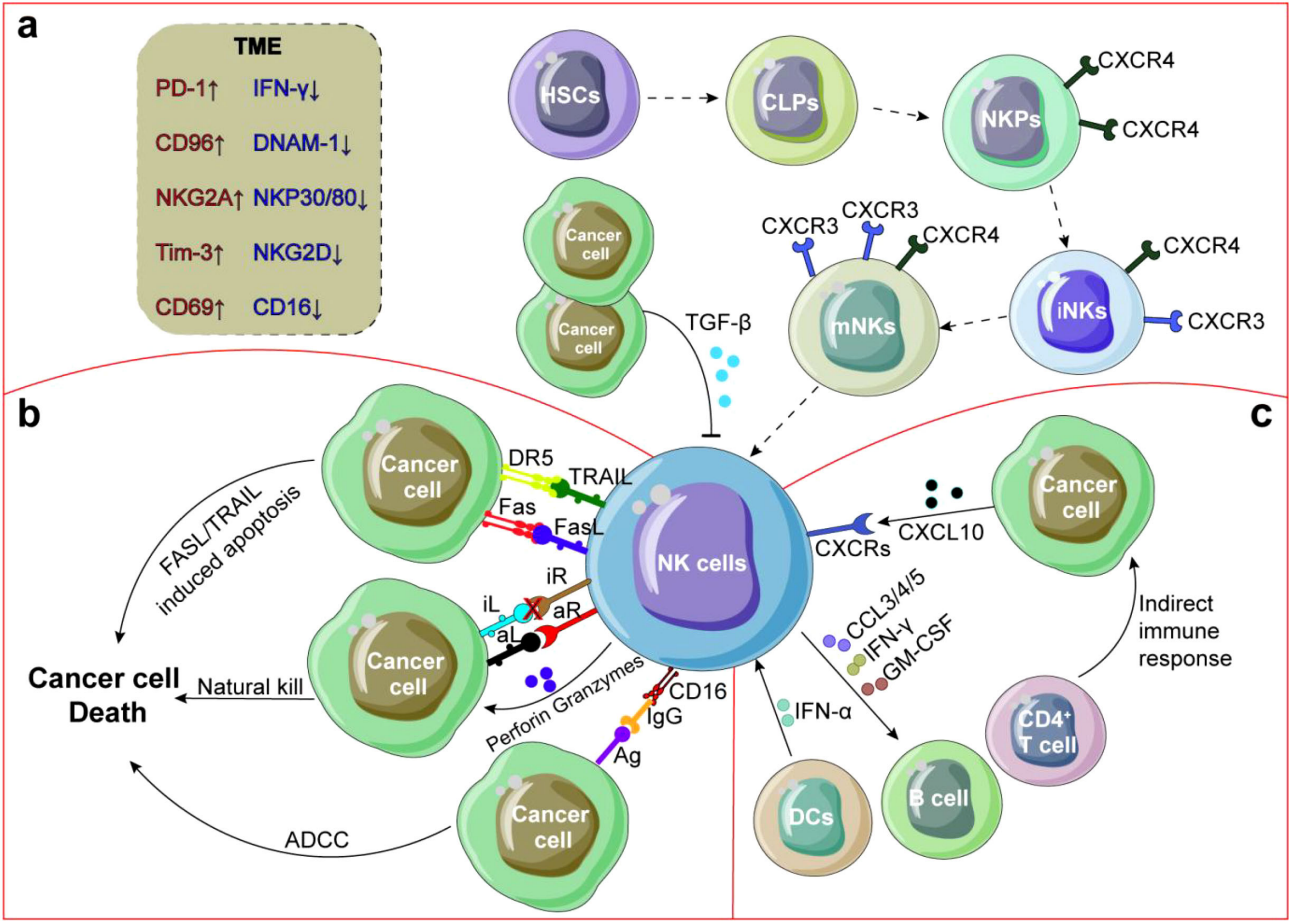
Crosstalk between NK cells and TME. **(a)** NK cells develop from HSCs in BM, with down-regulation of CXCR4 expression, upregulation of CXCR3 expression, NK cells mature and enter PB and tissues. **(b)** After exudation from PB, NK cells are recruited to TME by integrins, chemokine receptors and selectins, and secrete cytokines or chemokines to recruit other immune cells and improve anti-tumor response through degranulation, ADCC or FASL/TRAIL induced tumor cell apoptosis. **(c)** NK cell function is often inhibited by suppressors secreted by tumor cells and DCs or by direct interaction with CD4^+^ T cells. In addition, NK cells can also secrete angiogenic factors to promote tumor angiogenesis.

After differentiation, iNKs and some mNKs migrate from the parenchyma to the blood sinuses and eventually into the bloodstream, and then into secondary lymphoid tissue to further differentiate (60, 61). Some special subpopulations of NK cells return to BM to perform specific functions, such as monitoring and controlling infected cells (62). In mice, CD62L is necessary for NK cell homing (63). Factors that control NK cell trafficking and homing include integrins (64), selectin (65, 66), chemokine receptors and ligands (67, 68). For example, during NK cell maturation, the up-regulation of S1P5 and CX3CR1, concurrent with down-regulation of CXCR4, facilitates NK cells egress from BM into the bloodstream (69) (**Figure 1a**). Integrin and chemokine receptors, along with corresponding ligands or chemokines (e.g., VLA-4, CCL3, CXCR6, CCR5, CCL25-CCR9), are typically responsible for recruiting NK cells into peripheral tissues (37, 70, 71).

NK cell activation is governed by the dynamic equilibrium between activating and inhibitory signals (72), mediated through the engagement of their respective receptors with cognate ligands expressed on target cells (73, 74). In addition to their direct cytotoxic functions, NK cells can execute antibody-dependent cell-mediated cytotoxicity (ADCC) through the membrane receptor CD16 (75), inducing tumor cell apoptosis pathways via Fas ligands (FasL) or TNF-related apoptosis-inducing ligands (TRAIL) (76, 77) (**Figure 1b**). However, NK cell-based immunotherapy has not yet achieved optimal clinical outcomes due to NK cell exhaustion-a double-edged sword involving the homeostasis disorder dysregulation among NK cells, cytokines and TME (**Figure 1c**). This review highlights NK cells as dynamic integrators of TME signals, with combinatorial approaches targeting metabolic, epigenetic, and stromal factors offering new therapeutic avenues.

## 3 The relation between NK cells and AML

### 3.1 Characteristics of AML

AML arises from sequential somatic mutations in a primitive multipotential hematopoietic cell, which is a leukemia subtype with the highest incidence rate, poor outcomes (78, 79). The primary risk factors for AML include obesity, radiation exposure, prolonged exposure to high concentrations of benzene, and chronic tobacco smoke inhalation (80). A small but increasing proportion of AML cases (7% ~ 8%) develop after a patient with lymphoma, or an autoimmune disorder undergoing intensive chemotherapy, especially with alkylating agents platinum derivatives, or topoisomerase II inhibitors (81). According to French-American-British (FAB) classification (82), due to the origin and maturity of the leukemia cells, AML is generally divided into eight subtypes (M0-M7) (82).

LSCs are the initiation cells of AML, which have the ability of self-renewal and multidirectional differentiation to maintain the growth and recurrence of AML (83). LSCs are highly dependent on oxidative phosphorylation and glycolysis contributing to the aggressiveness of AML and are more drug-resistant (83–85). The onset of AML is closely related to gene mutations, such as NPM1, FLT3, IDH1/2, TP53 (86–88), as well as chromosomal rearrangements such as t(8;21) and inv16 (89, 90). AML development and progression are associated with dysregulated immune responses and induction of an immunosuppressive TME (91). Furthermore, AML blasts can hide from immune recognition by promoting T cell exhaustion and expansion of T regulatory cells (Tregs) (91). AML blasts have been found to increase the number of myeloid suppressor cells (MDSCs), polarize macrophages toward a pro-tumoral phenotype, and hamper NK cell effector functions (91). Moreover, AML cells can dysregulate the innate immune response by releasing cytokines and soluble factors or through direct contact with innate immune cells (92). In short, conventional dendritic cells (cDCs) are diminished in the AML BM compared to healthy donors, which may contribute to the lack of CD8^+^ T cells in the TME (93, 94) and increase the proportion of Tregs, MDSCs (95), which promotes the proliferation and metastasis of AML cells. BM stromal cells (BMSCs), specifically, CD73^+^CD105^+^CD271^+^ BMSCs subgroup in AML TME can promote the survival and proliferation of AML cells by secreting growth factors, thereby reducing treatment efficacy (96, 97).

Despite significant advances in conventional therapeutic approaches, including chemotherapy (e.g., cytarabine, anthracycline antibiotic doxorubicin) (98), targeted therapy (e.g., gilteritinib targeting FLT3 mutations) (99), radiation therapy (100), and HSCs transplantation (HSCT) have acquired great success in AML treatment (101), the overall survival rate of AML patients remain suboptimal, with 5-year survival rates ranging from 30% to 40% according to different studies (102–104). Moreover, while T cell-based immunotherapeutic approaches have shown promise in other hematologic malignancies, their efficacy in adult AML has been disappointingly limited, with pediatric trials only in the initial phases (105, 106).

Taken together, the treatment of AML is a long-term and complex process, and the current treatment plan has a certain effect on the alleviation of AML, but is far from achieving the goal of preventing recurrence or even complete cure. Therefore, the development of novel AML treatment methods have important clinical significance.

### 3.2 Treatment of AML based on NK cells

DNA hypomethylation drugs, currently FDA-approved for AML treatment, demonstrate interesting mechanistic effects: treatment with azacytidine and decitabine for 48 hours could decrease shedding of MICA, MICB, and ULBP2, consequently restoring NK cell function (107). Glycogen Synthase Kinase 3 Beta (GSK3β) expression is elevated in AML-NK cells and GSK3β pharmacological inhibition promotes conjugator formation by up-regulating LFA expression on NK cells and inducing ICAM-1 expression on AML cells, thereby enhancing the cytotoxic activity of AML-NK cells. This process only requires a short *ex vivo* exposure (16 hours) to 30 μM GSK3β inhibitors (SB415286, LY-2090314, Tideglusib) (108). Overactivation of NK cells by targeting GSK3β may be a novel strategy for the treatment of AML.

The recovery of NK cell functions after allo-HCT has been associated with protection against AML relapse (109) and NK cells have been identified as crucial players in the eradication of AML (110). Furthermore, donor NK cells, along with T cells, play a role in the graft-versus-leukemia (GVL) effect following HSCT for AML (111). In a phase I clinical trial (NCT01898793) (**Table 1**), adoptive transfer cytokine induced memory like NK (CIML-NK) cells (dose range: 0.5 ~ 10×10^7^ cells/kg) proliferated and expanded in patients with AML and observed in 5 of 9 evaluable patients, including 4 complete responses (112). Another recent phase II clinical study (NCT02782546) (**Table 1**) involved 15 AML patients who received consolidation therapy, followed by haploidentical HSCT and infusion of CIML-NK cells (dose level: 0.5 ~ 10×10^6^ cells/kg) and 13 patients (87%) achieved composite complete response after 28 days, the median event-free survival for all patients was 3.2 months, and 29% of the participants remained alive after 1 year (113). Another clinical trial (NCT03068819) conducted in 2021 (**Table 1**), Jeffrey J Bednarski used donor derived CIML-NK cell to treat 8 pediatric and young adult AML patients with HSCT recurrence found that 4 patients achieved complete response CIML-NK cell (dose level: 4 ~ 6×10^6^ cells/kg) infusion. Interestingly, a patient showed sustained remission during a 2-year follow-up after CIML-NK cell infusion without any subsequent treatment (114).

**Table 1:** Clinical application of NK cells in treating myeloid leukemia*.

NCT number *Disease*	Status *Phases*	Interventions *Sponsor*	Last update posted
NCT06201247 *R/R AML*	Recruiting *Phase 1*	CD123 CAR-NK *Peking University People’s Hospital*	2024
NCT05834244 *AML*	Recruiting *Phase 1*	Allogeneic NK cells infusion *M.D. Anderson Cancer Center*	2024
NCT06307054 *R/R AML*	Recruiting *Phase 1*	CLL-1 CAR-NK *Shanghai General Hospital, Shanghai Jiao Tong University School of Medicine*	2024
NCT06367673 *R/R AML*	Recruiting *Phase 1*	CLL-1 or CD33 iPSC-NK cells *Zhejiang University*	2024
NCT06138587 *AML*	Recruiting *Phase 1*	CIML NK cells infusion *Dana-Farber Cancer Institute*	2024
NCT03300492 *AML*	Recruiting *Phase 1/2*	NK-DLI infusion *University Hospital, Basel, Switzerland*	2024
NCT00720785 *CML*	Completed *Phase 1*	NK cells infusion *NHLBI*	2024
NCT02727803 *AML, CML*	Recruiting *Phase 2*	Allogeneic NK cell line NK-92 *M.D. Anderson Cancer Center*	2024
NCT05400122 *AML, CML*	Recruiting *Phase 1*	NK cells infusion *Jennifer Eva Selfridge*	2024
NCT06325748 *AML*	Recruiting *Phase 1*	CD33 and/or FLT3 CAR-NK *Senti Biosciences*	2024
NCT01904136 *AML, CML*	Completed *Phase 1/2*	NK cells infusion *M.D. Anderson Cancer Center*	2024
NCT05503134 *R/R AML*	Recruiting *Phase 1/2*	Universal Donor NK cells infusion *Nationwide Children’s Hospital*	2024
NCT05734898 *R/R AML*	Recruiting *NA*	NKG2D CAR-NK *Zhejiang University*	2023
NCT05744440 *R/R AML*	Recruiting *Phase 1*	Allogenic NK cells *Xuzhou Medical University*	2023
NCT06027853 *AML*	Recruiting *Phase 1*	CLL-1 CAR-NK *Zhejiang University*	2023
NCT05987696 *R/R AML*	Not yet recruiting *Phase 1*	CD33/CLL-1 dual CAR-NK, CD33 CAR-NK *Institute of Hematology & Blood* *Diseases Hospital, China*	2023
NCT04166929 *R/R AML*	Terminated *Phase 2*	CD3^-^CD56^+^ NK cells infusion *Fondazione Policlinico Universitario* *Agostino Gemelli IRCCS*	2023
NCT05665114 *R/R AML*	Recruiting *Phase 1*	Allogeneic NK cells infusion *Zhejiang University*	2023
NCT03068819 *AML*	Recruiting *Phase 1/2*	CIML NK cells infusion *Washington University School of Medicine*	2023
NCT06006403 *R/R AML*	Recruiting *Phase 1/2*	CD123 CAR-NK *Chongqing Precision Biotech Co., Ltd*	2023
NCT05256277 *R/R AML*	Terminated *Phase 1*	CIML NK cells *Zhejiang University*	2023
NCT05665075 *R/R AML*	Recruiting *Phase 1*	Allogeneic CD33 CAR-NK cells *Zhejiang University*	2023
NCT02890758 *AML, CML*	Completed *Phase 1*	NK cells infusion *Brenda Cooper, MD*	2023
NCT01823198 *AML, CML*	Completed *Phase 1/2*	CD56^+^CD3^-^ NK cells infusion *M.D. Anderson Cancer Center*	2023
NCT04836390 *AML*	Invitation *Phase 2*	Donor-derived ex-vivo expanded NK cells infusion *Michael Pulsipher, MD*	2023
NCT03349502 *R/R AML*	Completed *Phase 2*	Allogeneic NK cells infusion *Seoul National University Hospital*	2023
NCT05215015 *AML*	Unknown *Phase 1*	CD33/CLL-1 CAR-NK *Wuxi People’s Hospital*	2022
NCT05563545 *R/R ALL*	Completed *Phase 1*	CD19 CAR-NK *Shanghai Simnova Biotechnology Co.,Ltd.*	2022
NCT05008575 *R/R AML*	Unknown *Phase 1*	CD33 CAR-NK *Xinqiao Hospital of Chongqing*	2022
NCT05601830 *AML-MRD*	Recruiting *Phase 1*	Allogeneic NK cells infusion *Institute of Hematology & Blood* *Diseases Hospital, China*	2022
NCT04632316 *AML*	Unknown *Phase 1/2*	oNKord^®^ infusion *Glycostem Therapeutics BV*	2022
NCT03669172 *AML*	Completed *Phase 1/2*	CD56^+^CD3^-^ NK cells infusion *Martín, José Luis Díez, M.D.*	2021
NCT00703820 *AML*	Completed *Phase 3*	NK cells infusion *St. Jude Children’s Research Hospital*	2021
NCT01787474 *R/R AML*	Completed *Phase 1*	MBIL-21 expanded NK cells infusion *M.D. Anderson Cancer Center*	2021
NCT02763475 *AML*	Completed *Phase 2*	CD3^-^CD56^+^ NK cell infusion *M.D. Anderson Cancer Center*	2020
NCT01619761 *AML, CML*	Unknown *Phase 1*	NK cells infusion *M.D. Anderson Cancer Center*	2020
NCT02316964 *R/R AML*	Completed *Phase 1*	NK cells infusion *Sumithira Vasu*	2020
NCT02781467 *R/R AML*	Terminated *Phase 1*	Human cord blood derived, culture expanded NK cells infusion *Celularity Incorporated*	2020
NCT00789776 *AML, CML*	Completed *Phase 1/2*	NK cells infusion *Fred Hutchinson Cancer Center*	2020
NCT00582816 *R/R AML*	Terminated *Phase 1/2*	NK cells selected DLI *University of Wisconsin, Madison*	2019
NCT02123836 *AML*	Unknown *Phase 1*	NK cells infusion *National University Hospital, Singapore*	2019
NCT02809092 *AML*	Unknown *Phase 1/2*	NK cells infusion *Hospital de Clinicas de Porto Alegre*	2019
NCT03348033 *CML*	Unknown *Phase 1/2*	NK cells infusion *Hospital de Clinicas de Porto Alegre*	2019
NCT02477787 *AML*	Terminated *Phase 2*	Allogeneic, donor-derived NK cells *Asan Medical Center*	2019
NCT01947322 *AML*	Completed *Phase 1/2*	Allogenic CD3^-^CD56^+^ NK cells infusion *Assistance Publique-Hôpitaux de Paris*	2017
NCT00303667 *AML*	Completed *Phase 1/2*	CD3^-^CD19^-^ selected NK cells *Masonic Cancer Center*, *University of Minnesota*	2017
NCT00354172 *AML, CML*	Terminated *Phase 2*	NK cells infusion *Masonic Cancer Center, University of Minnesota*	2017
NCT00450983 *AML*	Terminated *Phase 2*	NK cells infusion *Fred Hutchinson Cancer Center*	2017
NCT01390402 *CML*	Completed *Phase 2*	NK cells infusion *M.D. Anderson Cancer Center*	2016
NCT02944162 *R/R AML*	Unknown *Phase 1/2*	CD33 CAR-NK cells *PersonGen BioTherapeutics Co., Ltd.*	2016
NCT02742727 *AML*	Unknown *Phase 1/2*	CD7 CAR-pNK cells *PersonGen BioTherapeutics Co., Ltd.*	2016
NCT00526292 *AML*	Completed *Phase 2*	NK cells infusion *Memorial Sloan Kettering Cancer Center*	2016
NCT00402558 *AML*	Completed *Phase 1*	Alloreactive NK cells infusion *M.D. Anderson Cancer Center*	2015
NCT01795378 *AML*	Completed *Phase 1/2*	Donor NK cells infusion *Asan Medical Center*	2015
NCT00640796 *R/R AML*	Completed *Phase 1*	Haploidentical donor derived NK cells infusion *St. Jude Children’s Research Hospital*	2014
NCT01220544 *AML*	Unknown *Phase 1/2*	Haploidentical transplantation with CD56^+^CD3^-^NK cells *Charite University, Berlin, Germany*	2010

*, Data from https://clinicaltrials.gov/; R/R, Relapsed/Refractory.

**Figure 2:**
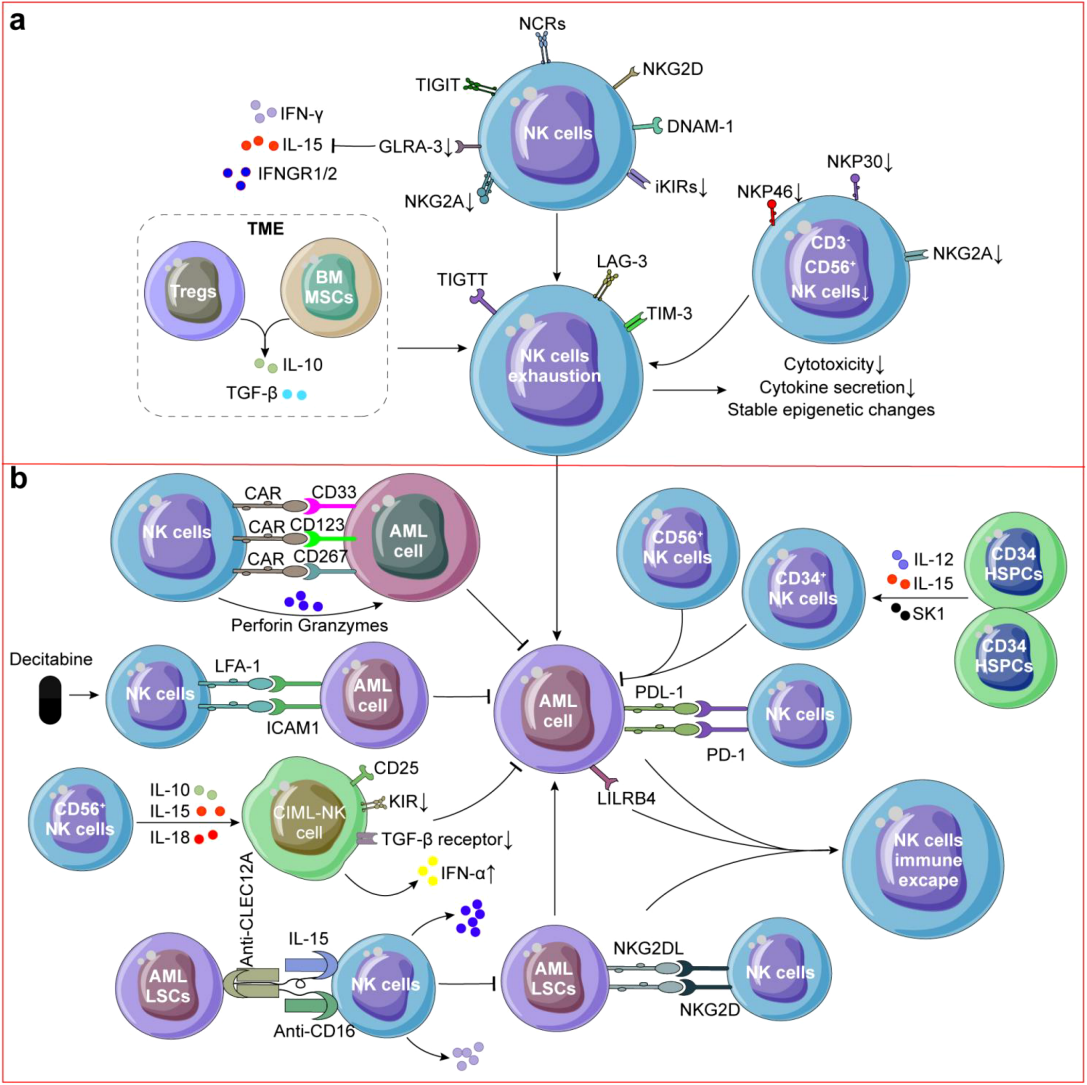
NK cells in AML. **(a)** NK cells exhibited impaired killing ability and exhausted phenotype. CD3^-^CD56^+^ NK cells in AML was decreased and Tregs or BMSCs secreting IL-10 or TGF-β also contributed to NK cell exhaustion in AML. **(b)** Decitabine could upregulating LFA expression on NK cells and inducing ICAM-1 expression on AML cells, thereby enhancing the cytotoxic activity of AML-NK cells. CD34^+^ NK cells, CD56^+^ NK cells or CIML-NK cells could directly kill AML cells. CD33/CD123/CD267 CAR-NK cells and TriKE molecules targeting CLEC12A were also used to reactive NK cells for killing AML cells and LSCs. Immune escape of NK cells is caused by residual LSCs and binding of PD-L1 on the surface of AML cells to PD-1 on the surface of NK cells, which results in AML recurrence.

CAR-NK cell therapy offers a promising therapeutic approach for treating AML. Primary CD33-targeting CAR-NK cells strongly reduce the burden of leukemia and prevents BM transplantation of leukemia cells without significant side effects (115, 116) (**Figure 2b**). For example, AML clearance in OCI-AML2-engrafted NSG-SGM3 mice was enhanced by injecting a total of three doses of 1 × 10^7^ of CD33 CAR-NK cells (115). The clinical trial, NCT05008575, currently underway at the Hematology Department of Chongqing Xinqiao Hospital in China targets leukemia cells expressing CD33, employing CAR-NK cells in combination with chemotherapy drugs (**Table 1**). Among the 10 evaluated patients, only 1 developed grade II CRS and no higher grade CRS occurred. Regarding anti-leukemia efficacy, 60% (6/10) of patients achieved complete remission 28 days after CAR-NK cell infusion (117, 118). In a preclinical study, CD123 CAR-NK cells (5-day OS: 100%) also showed lower acute toxicity than CD123 CAR-T cells (5-day OS: 0%) in a mouse model of transplanted artificial blood cells, while their anti-leukemia efficacy was comparable in a mouse model of AML (119).

Other studies have investigated novel therapeutic strategies to kill AML cells by combination of NK cells, and trispecific killer engager (TriKE) molecules targeting CLEC12A, the AML mice treated with CLEC12A TriKE had significantly less tumor burden compared to tumor alone or tumor with NK cells (120) (**Figure 2b**). Killer immunoglobulin-like receptor-human leukocyte antigen (KIR-HLA) mismatched NK cells (median, 29 × 10^6^/kg NK cells) infusions shown a significant expansion of KIR-mismatched NK cells (median, 5,800/mL of blood on day 14) could decrease relapse rates without increasing mortality in children with AML (121). CD276 (B7-H3) is highly expressed in leukemia cells of AML patients, FC optimized CD276 mAb specifically binds to CD276 on primary AML cells, promoting activation and enhancing anti-AML effects of NK cells (122).

On the whole, preclinical studies using NK cells to treat AML have shown encouraging results. However, challenges remain in ensuring long-term sustainability and mitigating potential off-target toxicity. Future clinical trials will determine the true potential of NK cells to revolutionize the AML treatment landscape.

### 3.3 NK cell exhaustion in AML

NK cells in AML patients mainly exhibit impaired killing ability and overall functional impairment of exhausted phenotype (**Figure 2a**) that indicates poor prognosis and high recurrence (123). On the one hand, the content of resident CD3^-^CD56^+^ NK cells in AML was decreased compared to healthy donors (124) (**Figure 2a**). NK cells from AML patients typically express more NCRs/NKG2D/DNAM-1, down-regulated NKG2A/iKIRs (113) and the ability of NK cells secreting IFN-γ is significantly reduced which may limit their ability to recognize and clear AML cells and patients with NK cell spectrum defects increased recurrence risk (*p* = 0.03) without regard for their cytogenetic classification (125). Turk et al. found that down-regulation of renin-angiotensin system (Ras) genes and neurotransmitter genes were involved in NK cell dysfunction in AML (126). For example, ATP6AP2 may modulate NK cell responses through regulation of pH homeostasis, autophagic flux, and NLRP3 inflammasome activation (127, 128). And, down-regulation of arginine may also impair cytokine secretion capacity of NK cells (129). Transcriptomic analysis of BM NK cells from AML patients reveals stress-induced inhibition of NK cell effector function. While CD160 expression is down-regulated in these NK cells, patients with CD160^high^ NK cells demonstrate significantly improved survival rates (130).

On the other hand, reduction in the number and function, high expression of T cell immunoglobulin and ITIM domain (TIGIT) of NK cells has been implicated in myelodysplastic syndrome (MDS), a condition that can progress to AML (131, 132). Through intercellular contact or secrete IL-10 or TGF-β by Tregs in AML TME contributed to NK cell exhaustion, which accelerates the occurrence of AML (22) (**Figure 2a**). And, AML development has been linked to impairing the BM homing capacity of infused NK cells (133). Unconventional CD56^-^CD16^+^ NK cells which decreased expression of NKG2A/NKp30/NKp46 in AML has been observed, indicating adverse clinical outcome (134, 135) (**Figure 2a**), and high expression of NKp46 contribute to the prognosis of AML patients after allo-HSCT (135). Similarly, the results were also found in animal experiments, in the MLL-AF9-induced mouse AML model, more mNKs were detected, but their mature state may not be sufficient to fully exert their anti-AML effects (136).

In summary, NK cell exhaustion promotes the occurrence, progression, and recurrence of AML. Increasing the number of NK cells or activating their function can contribute to the treatment and prognosis of AML. Therefore, NK cell-based treatment strategies for AML show theoretical promise and warrant further investigation.

### 3.4 Immune evasion of NK cells in AML

Significant challenges persist in maximizing the therapeutic potential of NK cells in AML treatment, particularly as AML cells have developed sophisticated mechanisms to evade NK cell-mediated immunosurveillance (137). Claudia Lengerke et al. demonstrated that NKG2D ligands are generally expressed on most of AML cells but not on LSCs, potentially explaining their resistance to NK cell-mediated cytotoxicity and their role in therapeutic resistance (138). In AML, the function of NK cells can be inhibited by various factors, including the presence of multiple immunosuppressive factors in the TME, such as TGF-β and IL-10 (**Figure 2a**), which inhibit the activity and proliferation of NK cells (139). In AML, Tregs and BMSCs typically expand and secrete inhibitory cytokines to reduce the anti-AML activity of NK cells (140, 141). AML cells may express inhibitory ligands such as PD-L1 (**Figure 2b**), which can bind to receptors on the surface of NK cells and inhibit their activity (142, 143). Some AML cells can induce NK cell death by activating the apoptotic pathway (144, 145) and overexpressing inhibitory immune molecules LILRB4 (**Figure 2b**), directly inhibiting the activity of NK cells (137, 146). AML cells can alter TME, for example, tumor associated fibroblasts may suppress NK cell function by secreting inhibitory factors (147, 148). One study suggested that combination of hypomethylating agents and NK cell infusion could be a promising strategy to overcome AML immune escape (137). Although there has been progress in understanding the immune escape mechanism of NK cells in AML, there are still some unknown areas.

Future research directions in AML should prioritize: i) elucidating the molecular mechanisms underlying AML-mediated NK cell dysfunction; ii) characterizing the dynamic interactions between NK cells and the leukemic microenvironment; iii) developing patient-stratified NK cell-based immunotherapeutic approaches to optimize clinical outcomes while minimizing adverse effects.

## 4 The relation between NK Cells And CML

### 4.1 Characteristics of CML

CML is a multipotential hematopoietic stem cell disease (149). The hematopoietic cells contain a reciprocal translocation between chromosomes 9 and 22 in more than 90% of patients with classic morphologic findings, which leads to an overtly foreshortened long arm of one of the pair of chromosome 22, referred to as the Philadelphia chromosome (Ph) (150). The most iconic change in CML is Ph, caused by the reciprocal translocation t(9;22) (q34;q11.2), resulting in the formation of BCR-ABL fusion gene which was a clinical diagnostic marker (151). The chronic myelogenous leukemias (CMLs) include BCR rearrangement-positive CML, atypical CML, chronic myelomonocytic leukemia, juvenile myelomonocytic leukemia, chronic neutrophilic leukemia, chronic eosinophilic leukemia, and chronic basophilic leukemia (152).

Similar to AML, LSCs are the main cause of CML occurrence and recurrence. CML is caused by activation of BCR-ABL in HSCs and converting them into LSCs defined as CD34^+^CD38^-^ lead to expansion of myeloid progenitors (153). For example, in a phase 2 pilot study of 46 CML patients, patients who did not achieve major molecular response (MMR) at 18 months of treatment with imatinib or dasatinib had Ph^+^ cells (> 75%) in the CD34^+^CD38^-^ fraction (154). Tyrosine kinase inhibitors (TKIs) induce up-regulation of N-cadherin in LSCs and adhesion to MSCs leads to activation in typical Wnt signaling, protects LSCs from apoptosis and promotes relapse (155). LSCs may also avoid eradication by modulating host immune surveillance in TME, and cytotoxic T lymphocytes (CTLs) fail to induce an appropriate immune response against CML cells through CTLs exhaustion due to the interaction of the PD-1 receptor expressed on CTLs with the inhibitory ligand PD-L1 expressed on CML cells (156). Meanwhile, the number and activity of CD4^+^ helper T cells and CD8^+^ cytotoxic T cells are decreased in CML patients, manifested by up-regulation of surface markers such as PD-1 and CTLA-4, ultimately leading to a weakened immune response to CML cells (157). The expression of Lin^-^CD11b^+^CD33^+^ MDSCs was increased at diagnosis (38.6 ± 6.5%) compared with MMR (11.8 ± 2.5%, *p* = 0.0004) and CD4^+^CD25^high^CD127^-^Foxp3^+^ Tregs was higher at diagnosis (2.3 ± 0.2%) compared with MMR (1.8 ± 0.2%, *p* = 0.02) (158) indicating the treatment of CML may require a combination of TKI and immunotherapy.

TKIs significantly improve patient outcomes by specifically inhibiting the activity of BCR-ABL tyrosine kinase such as asciminib, imatinib, nilotinib, dasatinib, etc (159). The advancement of TKIs has substantially prolonged the survival time of most CML patients, with 5-year survival rates reaching approximately 65% to 70% (160). Patients taking imatinib showed that about 86% to 93% were still alive 5 years (161). However, long-term TKI therapy may result in drug resistance in approximately 15 ~ 17% of cases, primarily due to T315I mutations and LSCs activation, presenting an urgent clinical challenge (162). HSCT, interferon therapy (Pegylated interferon), hormone treatment, radiation therapy were also used to treat CML patients, but recurrence of CML still occur because the immune system of patient is not durably reactivated. In order to solve the above problems, CD19 (163), CD26 (164) or CD38 (165) CAR-T was used to overcome TKIs and chemotherapy resistance which achieved satisfactory results. However, it is worth noting that there was some extratumoral cytotoxicity towards activated lymphocytes (164).

As described above, for the clinical treatment of CML and the reversal of chemotherapy resistance, it is a feasible strategy to combination of chemotherapy novel immunotherapies with high efficiency and low toxicity.

### 4.2 Treatment of CML based on NK cells

**Figure 3:**
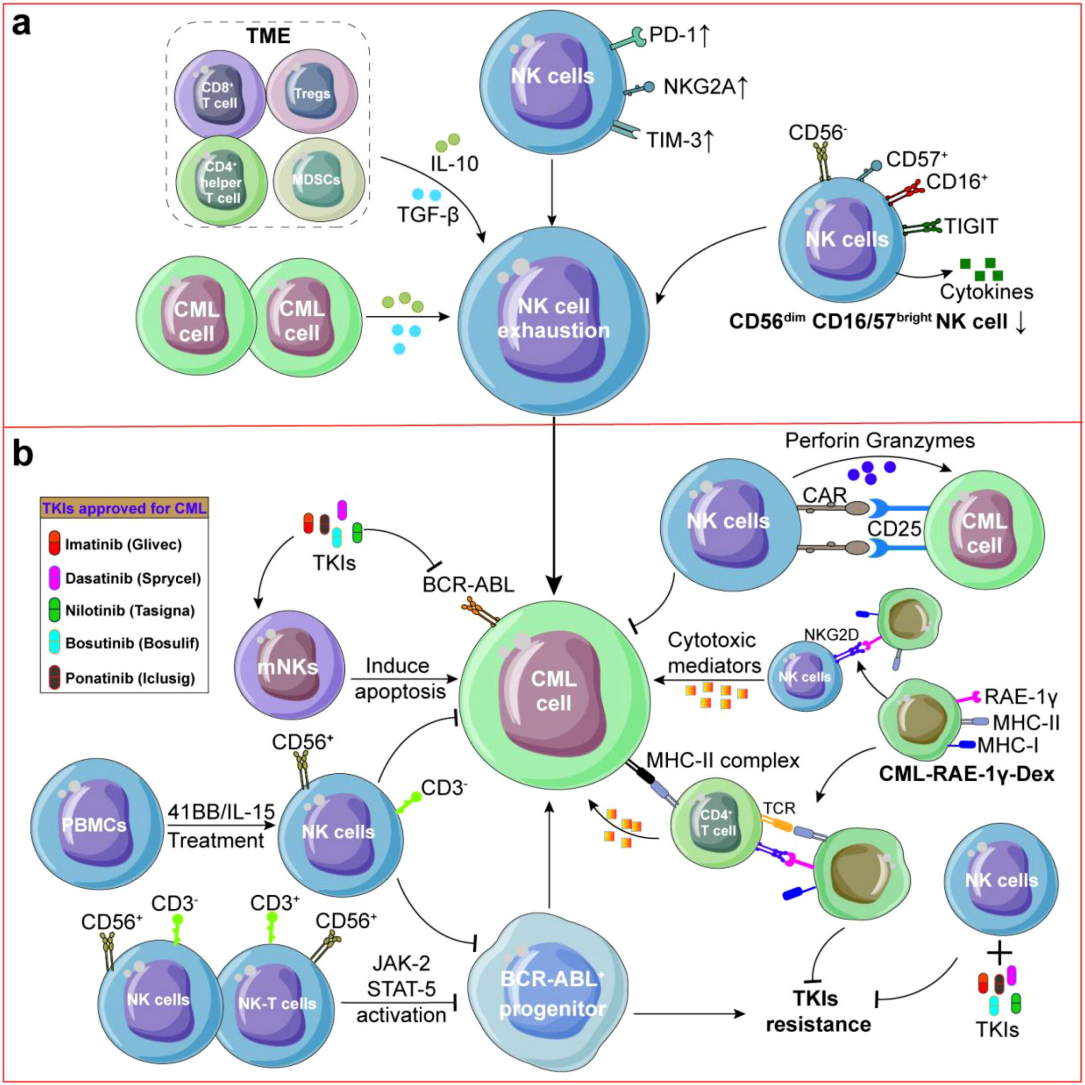
NK cells in CML. **(a)** Immune cell subpopulations and CML cells secreted IL-10 or TGF-β which contributed to dysfunction of NK cells. Meanwhile, Mature CD56^dim^CD16/57^bright^ NK cells and circulating NK cells were reduced which could promote CML occurence and recrudescence. **(b)** NK cells could demonstrate direct cytotoxicity against CML cells and both CD56^+^CD3^-^ NK cells and CD56^+^CD3^+^ NK-T cells suppress proliferation of BCR-ABL^+^ progenitors through JAK-2/STAT-5 pathway activation. To enhance NK cell expansion and cytotoxicity, PBMCs cells were treated with IL-15 and 41-BB could increase the number of CD56^+^CD3^+^ NK cells, CD25 CAR-NK92 cells can effectively kill CML cells. In reversing TKIs resistance, CML-RAE-1γ-Dex activate both NK cells and T lymphocytes, and inhibit the proliferation of TKIs-resistant CML cells.

NK cell-based therapeutic strategies for CML have garnered increasing attention in recent years (166). To enhance NK cell expansion and cytotoxicity, modified dendritic cell-derived exosomes activated NK cells could improve anti-CML effects via NKG2D/NKG2D-L pathway (167). K562 cells were modified by expressing a membrane-bound form of IL-15 which could induce higher expansion of CD56^+^CD3^-^ NK cells from PB (168, 169) (**Figure 3b**). Modification effects of IL-15 are also used in MM (170), MDS (171), colon cancer (CRC) and pancreatic cancer (PDAC) (172). Qi Li also reported that IL-21(50 ng/mL) could increase the number of CD56^+^CD3^+^ NK cells among PBMCs (173) revealing the feasibility of IL-15 or 21-secreting CAR-NK in treating CML. At present, CAR-NK based CML therapy is in the animal experimental stage (**Figure 3b**), Jusuf Imeri found that CD25 CAR-NK92 cells can effectively treat NSG mice transplanted with K562-CD25 cells and significantly increased the survival rate as compared to the untreated and NK92 WT treated cohorts (*p* < 0.01) (174) indicating that it is feasible to treat CML with CAR-NK.

Multiple clinical trials have been conducted exploring NK cell-based treatments for CML. For example, the clinical trial (NCT00720785) conducted in 2021 (**Table 1b**) verified the anti-CML effects by combination of Bortezomib and NK cells infusion. In another phase I/II study (NCT03348033) assessing the safety and feasibility of autologous activated and expanded NK cells in CML, infusion of NK cells significantly reduced BCR-ABL gene expression compared to untreated controls (**Table 1**). NCT02727803 and NCT02727803, both CML clinical trials, similarly demonstrated that infusion of the allogeneic NK cell line NK92 or primary CD56^+^CD3^-^ NK cells, respectively, significantly reduced BCR-ABL gene expression compared to controls (**Table 1**). These results provided a clinical reference for NK cell-based CML therapy and further confirmed its feasibility.

NK cells can be also used to reverse TKIs resistance. For reversing TKIs resistance, NK cell-derived extracellular vesicles (EVs) exhibit stronger cytotoxicity against imatinib resistant cells than parental ones by reducing the CD34^+^/CD38^-^ sub-populations (166). In advanced EGFR-mutated non-small-cell lung cancer, Ye Fei et al. found that combination of CD8^+^CD56^+^ NKT cells with gefitinib can overcome EGFR-TKIs resistance, revealing the universality of NK cells in reversing TKIs resistance (175). NK92-CD16 cells preferally kill TKIs-resistant cells by targeting ICAM-1, especially combination with cetuximab (an EGFR-targeted mab), the effect of reversing resistance is further enhanced (176, 177). CML-specific Dendritic cell-derived exosomes (CML-RAE-1γ-Dex) rich in RAE-1γ activate both NK cells and T lymphocytes (**Figure 3b**), and inhibit the proliferation of imatinib-resistant CML cells with T315I mutations (167).

Future directions in CML should prioritize: i) optimization of NK cell expansion and activation protocols; ii) advancement of CAR-NK development and clinical validation; iii) longitudinal assessment of NK cell therapy safety and efficacy in CML patients through rigorous clinical trials.

### 4.3 NK cell exhaustion in CML

Most NK cells in CML were active CD56^dim^ cluster in PB and interacted with leukemia cells through inhibitory LGALS9-TIM3 and PVR-TIGIT interactions (178). Other studies also found the expression of TIGIT was increased on CD56^dim^ NK cells in CML PB while CD57 was increased on CD56^dim^ NK cells in CML BM (179), indicating that reversing immune suppression of PB NK cells by blocking TIGIT while improving proliferation of BM NK cells via targeting CD57 may be more effective in anti-CML (**Figure 3a**). Similar to AML, the absolute number of mature CD56^dim^CD16/57^bright^ NK cells and circulating NK cells in PB were significantly reduced in CML patients (180). For example, the percentage of CD56^+^ NK subset in total circulating NK pool was significantly reduced in 21 CML patients (2.5% ± 0.5%) compared with normal donors (5.7% ± 0.8%) (*p* < 0.001) (181). Meanwhile, NK cells were dysfunctional during CML progression from chronic phase to blast crisis because BCR-ABL decreased the natural cytotoxicity of NK cells and the acquisition of KIRs (182). BCR-ABL could also interference with NK cell differentiation and exogenous addition of BCR-ABL transduced autologous CD34^+^ cells could inhibit NK cell differentiation of normal umbilical cord blood CD34^+^ and CD38^-^ cells (183). Another study found that CML cells could effectively inhibit the cytotoxicity of baseline and IL-2-induced NK cells to K562 cells through reducing NADPH oxidase-mediated formation of ROS (184) revealing the complexity of interrelation between CML and NK cells.

NK cell exhaustion could promote CML recrudescence (**Figures 3a, b**). Firstly, inhibition of ROS can restore NK cell numbers and enhance their cytotoxicity against CML (185). And, knockout of *CXCR4* leads to NK cell depletion and TKIs resistance (186, 187). Secondly, both CD56^+^CD3^-^NK cells and CD56^+^CD3^+^ NK-T cells suppress granulocyte-macrophage colony formation in BCR-ABL^+^ progenitors through JAK-2/STAT-5 pathway activation, while sparing normal CD34^+^ cells (188). Meanwhile, CML cells may release MICA into plasma, leading to NKG2D down-regulation on CD56^+^ NK cells and subsequent NK cell dysfunction (188). Thirdly, hyper-functional adaptive-like NK cells in CML MMR patients exhibited a 56-fold expansion of a normally rare subset (*p* < 0.01) which diminished following TKIs resistance (189). Degranulation of NK cells can be partially saved by inhibiting CIS or TNF-β to overcome NK cell suppression (190). Consistent with these findings, Amandine Decroos et al. found that high frequencies of perforin-expressing NK cells is associated with treatment-free remission (191), and these results suggest that targeting inflammatory signals can enhance NK cell-based CML immunotherapy.

Clinical evidence demonstrates that CML patients with favorable responses to imatinib exhibit elevated levels of CD3^-^CD56^+^ NK cells (*p* = 0.0043), CD16^+^ NK cells (*p* = 0.0046) and CD57^+^ NK cells (*p* = 0.0208) (192). Moreover, NK cells from TKIs-treated CML patients show enhanced expression of NKp30/NKp46/NKp80 (193), suggesting that NK cell maturation status correlates with TKIs response. The clinical trial, NCT03239886, revealed that patients experiencing relapse after 6-month imatinib discontinuation demonstrated significantly lower NK cell proportions compared to non-relapsing patients, indicating NK cell levels as potential predictive biomarkers for molecular relapse risk (**Table 1**). In the context of allo-HSCT, patients receiving HLA-matched but KIR3DL1-mismatched transplants showed reduced BCR-ABL transcription levels and enhanced NK cell activity (194). Notably, KIR3DL1^+^ NK cells exhibited rapid recovery (17.1% median at days 28-56, increasing to 41.7-86.0% by days 28-41), suggesting that KIR3DL1-HLA-B interactions may modulate anti-tumor immunity.

The above studies indicate that NK cells play an important role in the occurrence and recurrence of CML and can be used as an effective weapon in the treatment of CML. Next, we will further summarize the mechanism of NK cell immune escape in CML.

### 4.4 Immune evasion of NK cells in CML

Immune escape represents a critical barrier to NK cell-based immunotherapy. The CML microenvironment is characterized by abundant MDSCs and Tregs (**Figure 3a**), which suppress NK cell activity and proliferation, facilitating immune evasion (195, 196). Changes in receptors on the surface of NK cells also mediate immune escape. Ya-Ching Hsieh et al. found that up-regulation of inhibitory receptors NKG2A on NK cells leads to loss of effective recognition of CML cells by NK cells (197), Meanwhile, the CML cells themselves can secrete a variety of cytokines such as IL-10 and TGF-β, which could inhibit the function and proliferation of NK cells (107). CML cells may also evade NK cell attacks by altering surface antigens, such as down-regulating the expression of NKG2DL or MHC, to inhibit the action of NK cells by secreting soluble MICA (sMICA) and reduce NK cell recognition (198). CML cells also inhibited NK cell activation signals by up-regulating TIM-3 or PD-1 that interact with receptors on the NK cell surface (178). Many CML cells exhibit a deficiency of the HLA-DR antigen, which is an important molecule for NK cells to recognize target cells (199). When HLA antigen expression decreases, the recognition ability of NK cells is limited (199). Future studies need to focus on how to restore the function of NK cells and prevent immune escape of NK cells and treatment failure.

## 5 Comparative analysis of CAR-T and CAR-NK cell therapy in AML/CML

NK cells present several advantages over T cells, including reduced toxicity (200), superior scalability in manufacturing (201), and an intrinsic ability of CAR-NK to differentiate between malignant and non-malignant cells (202). CAR-T cells mediate cytotoxicity through T-cell receptor (TCR) activation via CD3ζ signaling domains and costimulatory domains (e.g., 4-1BB/CD28), targeting specific antigens (e.g., CD19, CD22), but AML/CML lack ideal tumor-specific antigens, leading to on-target/off-tumor toxicity against normal HSCs (23, 203). The limitations of CAR-T cells in AML/CML mainly include: shared expression of targets (e.g., CD33, CD123, FLT3) on normal hematopoietic progenitors causes myelosuppression (203), and high antigen escape rates (> 60% in heterogeneous AML) (23, 203). The mechanisms of CAR-NK cells include: scFv-mediated antigen recognition (e.g., CD19, CD70), innate cytotoxicity (“missing-self” recognition of low MHC-I cells), CD16-mediated ADCC, and IFN-γ/TNF-α secretion (204–206). CAR-NK cells can eliminate AML HSCs with low MHC-I expression (feature of advanced CML) (207) and target CD70 (highly expressed on AML blasts and CML blast crisis), while also clearing alloreactive T cells to prolong persistence (208). The advantages of CAR-T cells in AML/CML are long persistence (> 12 months) and efficacy against high tumor burden (23). However, CAR-T cells require prolonged manufacturing (3–5 weeks), and the antigen density was low (23). In contrast, iPSC-derived CD70 CAR-NK cells have demonstrated > 90% AML cell clearance while suppressing alloreactive T-cell rejection (208). In short, compared with CAR-T cells, CAR-NK cells exhibit higher clinical efficacy and translational potential.

## 6 Conclusions

In the past 15 years, clinical trials of NK cell-based therapy for ML have expanded significantly with promising outcomes. Nevertheless, significant challenges remain, particularly in optimizing NK cell expansion, variability in response, circumventing immune surveillance mechanisms and manufacturing scalability. The future directions or therapeutic strategies of NK cell-based therapeutics in ML depends on the following critical factors. First, the diversification of cell sources, encompassing both autologous and allogeneic NK cells, along with established cell lines such as NK92, offers more therapeutic options. Next, concurrent advances in genetic engineering platforms, particularly CRISPR/Cas9 technology, are expected to enhance targeting specificity and anti-ML efficacy. Last, the integration of NK cell therapy with conventional treatments, including chemotherapy and radiotherapy, as well as other immunotherapeutic approaches, could yield superior therapeutic outcomes. Additionally, the development of patient-tailored NK cell products based on individual ML characteristics presents an opportunity to optimize therapeutic efficacy while minimizing adverse effects. In conclusion, the NK cell-based therapeutic strategy for ML demonstrates both theoretical soundness and clinical feasibility, warranting further research focused on advancing NK cell product development and clinical translation.
